# Prevalence of pancreatic cystic neoplasms on imaging exams: association with signs of malignancy risk

**DOI:** 10.1590/0100-3984.2017.0105

**Published:** 2018

**Authors:** Aline Falqueto, Gustavo Lemos Pelandré, Mariânges Zadrozny Gouvêa da Costa, Marcelo Souto Nacif, Edson Marchiori

**Affiliations:** 1 MD, Resident in Radiology and Diagnostic Imaging at the University Hospital of the Universidade Federal de Santa Catarina (UFSC), Florianópolis, SC, Brazil; 2 Assistant Professor of Radiology at the Universidade Federal de Santa Catarina (UFSC), Florianópolis, SC, Brazil; 3 Professor of Gastroenterology at the Universidade do Sul de Santa Catarina (Unisul), Palhoça, SC, Brazil; 4 Adjunct Professor in the Department of Radiology of the Universidade Federal Fluminense (UFF), Niterói, RJ, Brazil; 5 Full Professor of Radiology at the Universidade Federal do Rio de Janeiro (UFRJ), Rio de Janeiro, RJ, Brazil

**Keywords:** Pancreas, Pancreatic cyst, Pancreatic neoplasms

## Abstract

**Objective:**

To analyze the prevalence of cystic lesions of the pancreas on imaging exams and their association with signs of malignancy risk.

**Materials and methods:**

This was an observational cross-sectional study, in which we evaluated 924 sequential computed tomography and magnetic resonance imaging scans of the abdomen. For all of the patients included in the study, we reviewed the demographic data available in the medical records and evaluated the images.

**Results:**

Cysts were observed in 4.5% of patients, the prevalence of cysts being highest (7.6%) in patients over 60 years of age. Lesions were detected at higher rates on magnetic resonance imaging and in patients with pancreatic symptoms (6.1% and 42.9%, respectively). Signs of malignancy risk were observed in 26.3% of the patients, more frequently in those who were male and over 60 years of age.

**Conclusion:**

The prevalence of pancreatic cysts was 4.5%. Signs of malignancy risk were observed in 26.3% of the cystic neoplasms identified.

## INTRODUCTION

Cystic lesions of the pancreas comprise a broad spectrum of diseases with varied clinical features and prognoses. When arising from congenital syndromes or pancreatic insults (inflammation or trauma), they can have a non-neoplastic origin, one notable example being pseudocysts, which are often associated with pancreatitis^(1-3)^.

Pancreatic cystic neoplasms include those arising from the ductal epithelium (serous cystic neoplasm, mucinous cystic neoplasm, intraductal papillary mucinous neoplasm, and ductal adenocarcinoma), those arising from endocrine cells, those arising from acinar cells (acinar cell cystadenoma and acinar cell cystadenocarcinoma), and those arising from mesenchymal elements^(2,4)^. Ductal adenocarcinoma accounts for approximately 90% of malignant neoplasms and consists, in most cases, of moderately differentiated mucinous carcinomas originating from the cuboid epithelium of the pancreatic ducts. The annual incidence of ductal adenocarcinoma is estimated at more than 367,000 cases, more than 10,000 occurring in Brazil, with an overall mortality rate of 98%^(5)^.

With the technological advances in imaging examinations and their wider availability in recent decades, pancreatic cystic lesions have been diagnosed with increasing frequency as incidental findings in radiological studies of the abdomen, especially on computed tomography (CT) and magnetic resonance imaging (MRI) scans. Although the exact prevalence of such incidentally detected pancreatic cystic lesions is not known, their identification should not be ignored, because some will undergo malignant degeneration, especially in elderly patients, symptomatic patients, and patients with concomitant pancreatitis^(1,3,6,7)^.

The radiological characteristics, in isolation, do not always allow benign and malignant lesions to be distinguished with any degree of certainty^(3,8-10)^. According to the American Gastroenterology Association guidelines, the risk of malignancy is higher for cystic lesions > 30 mm with a solid component or in the presence of dilation of the main pancreatic duct, and such lesions should be submitted to pathological investigation^(11)^.

The objective of the present study was to determine the prevalence of pancreatic cystic lesions identified on CT and MRI scans. We also attempted to quantify associations between such lesions and risk factors for malignancy.

## MATERIALS AND METHODS

This was an observational cross-sectional study of patients seen at a private imaging facility collaborating with this study, predominantly providing outpatient care. The study was approved by the Research Ethics Committee of the Universidade do Sul de Santa Catarina (reference no. 1,122,097/2015).

We evaluated a sample comprising 1000 medical charts of adult patients undergoing CT and MRI examinations between January and May 2015. We calculated the sample size using a formula based on a prevalence study, with OpenEpi software, version 3.01, and the following parameters: a statistical power of 80% and a confidence level of 95%.

We included patients who underwent CT or MRI of the abdomen after January 1, 2015, selecting patients sequentially until 500 MRI scans and 500 CT scans had been obtained, that goal being met in May of the same year. Patients under 18 years of age were excluded, as were those with a history of manipulation of the pancreas. If a patient underwent more than one examination during the study period, only the most recent examination was included in the analysis. We reviewed the demographic data available in the medical charts, as well as the images, of all the patients included in the study. The following variables were analyzed: gender, age, symptoms, clinical indication, and type of health care plan.

The CT and MRI images were reviewed by a radiologist with six years of experience in abdominal radiology. Disagreements regarding the initial report were resolved by consensus among the authors. We analyzed the following aspects of the CT and MRI scans: number of cystic lesions, location, dimensions, presence of solid component, ductal dilation, calcifications, septations, parenchyma atrophy, peripancreatic inflammatory alterations, and the diagnostic impression of the radiologist. On the basis of the lesion morphology, radiological aspects, and the impression of the radiologist, the pancreatic cystic lesions were divided into two groups: neoplastic and non-neoplastic.

Statistical analysis was performed with Excel software and with the Statistical Package for Social Sciences version 18 (SPSS Inc., Chicago, IL, USA). Pearson's chi-square test was used in order to describe the associations with the qualitative variables. The level of significance was set at *p* < 0.05.

## RESULTS

Of the 1000 patients included in the study, 33 were under 18 years of age, 17 had undergone pancreatic resection, and 26 had undergone duplicate exams. The population studied therefore comprised 924 patients-558 females (60.4%) and 366 males (39.6%)-with a mean age of 55.2 years. Regarding the type of examination, 481 patients (52.1%) underwent CT, 91.0% of those patients receiving intravenous contrast. Of the 443 patients (47.9%) who underwent MRI, 92.1% received intravenous contrast. The vast majority of patients (88.2%) had supplementary health insurance plans.

**Figure 1:**
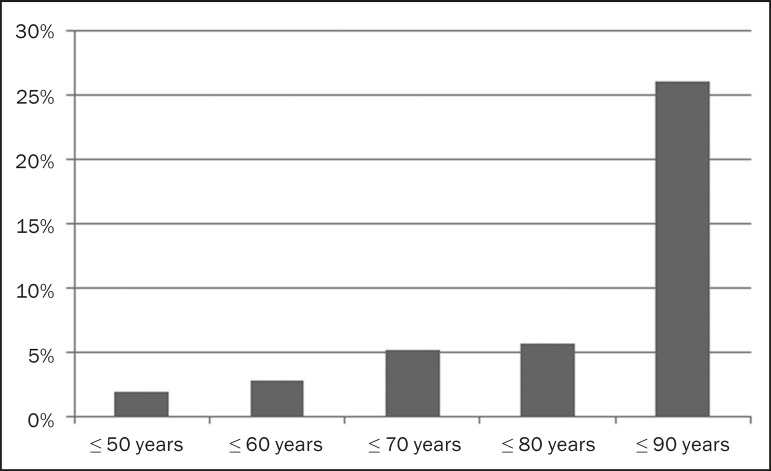
Prevalence of pancreatic cystic lesions, by age group.

The mean age of the patients in the sample was 67.9 years. Pancreatic cystic lesions were identified in 42 (4.5%) of the patients, the prevalence being similar in men and women (Table 1). The prevalence increased progressively with age (Figure 1). The prevalence was higher among patients with pancreatic complaints (42.9%). Pancreatic cystic lesions were detected in 6.1% of the MRI scans and in 3.1% of the CT scans (*p* < 0.05).

**Table 1:** Demographic characteristics of patients with and without pancreatic cysts.

	With cyst(s)	Without cyst(s)	
Characteristic	(n = 42)	(n = 882)	*P*-value
Gender			
Male	18 (4.9%)	348 (95.1%)	0.747
Female	24 (4.3%)	534 (95.7%)	
Age group			
18-39 years	1 (0.6%)	173 (99.4%)	< 0.001
40-59 years	12 (3.3%)	357 (96.7%)	
> 60 years	29 (7.6%)	352 (92.4%)	
Imaging modality			
MRI	27 (6.1%)	416 (93.9%)	0.039
CT	15 (3.1%)	466 (96.9%)	
Clinical indication			
Pancreatic complaint	21 (42.9%)	28 (57.1%)	< 0.001
Other complaint(s)	20 (2.4%)	809 (97.6%)	
None	1 (2.2%)	45 (97.8%)	

**Figure 2:**
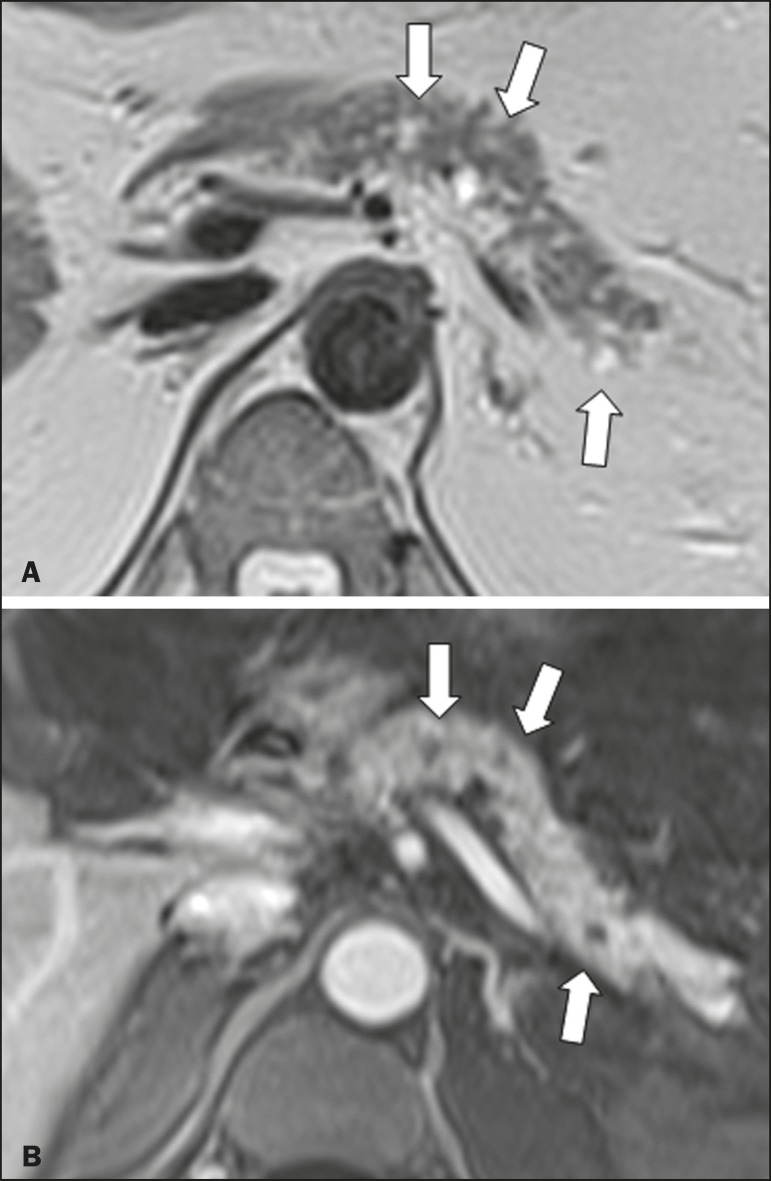
MRI scans. T2-weighted sequence (**A**) and contrast-enhanced T1-weighted sequence (**B**), showing small cystic lesions in contact with the pancreatic duct (arrows), with aspects characteristic of intraductal mucinproducing tumors.

**Figure 3:**
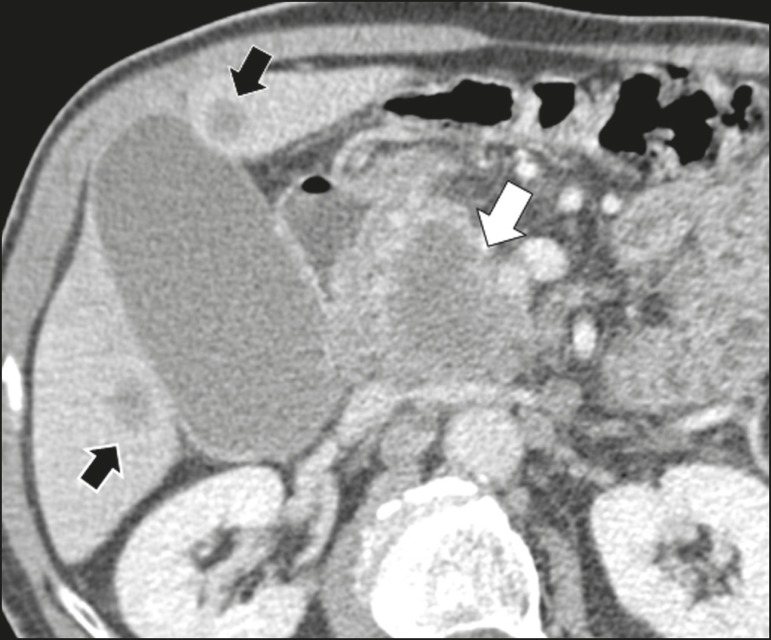
Contrast-enhanced CT scan showing a massive solid-cystic lesion in the head of the pancreas, measuring 40 mm (white arrow), together with liver lesions with aspects characteristic of secondary implants (black arrows). Appearance of pancreatic adenocarcinoma.

**Figure 4:**
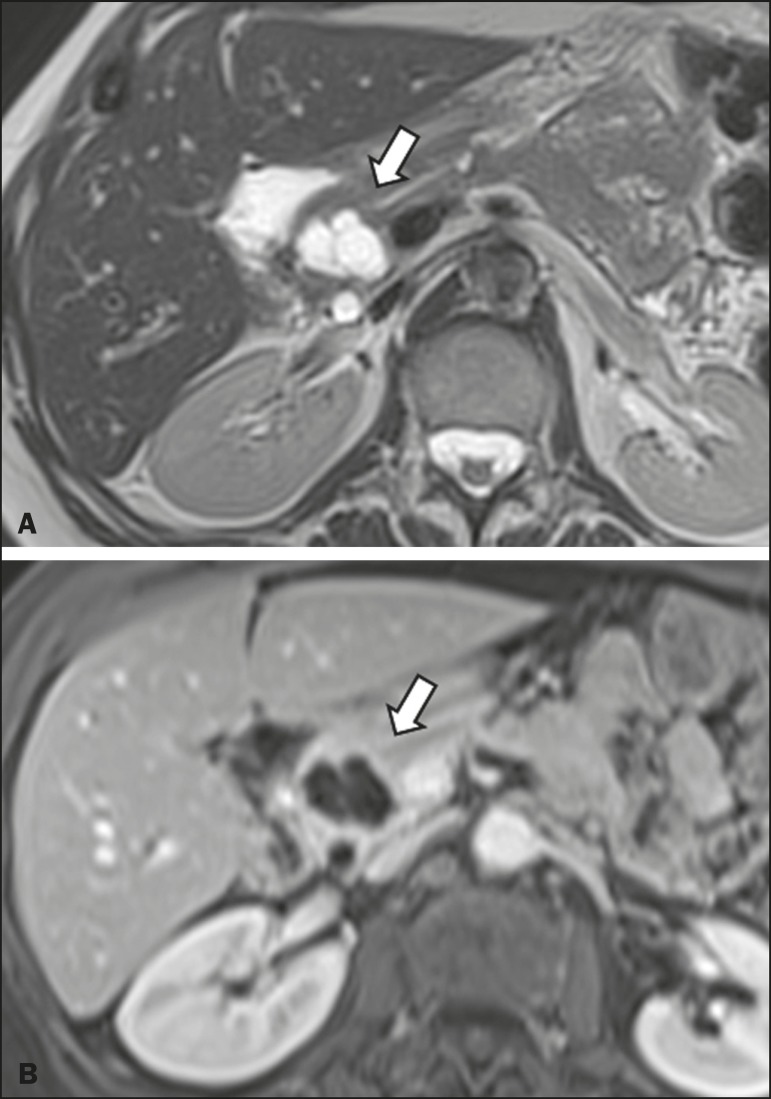
MRI scans. T2-weighted sequence (**A**) and contrast-enhanced T1- weighted sequence (**B**), showing a multilocular cystic lesion in the head ofthe pancreas, with fine septations and the appearance of a serous cystic neoplasm (arrows).

On the basis of the CT and MRI morphological findings, as well as the clinical data, 4 (9.5%) of the 42 cystic lesions detected were classified as non-neoplastic (pseudocysts) and 38 (90.5%) were classified as neoplastic. Of the cystic lesions classified as neoplastic, we categorized 31 (81.6%) as intraductal mucin-producing tumors (Figure 2), 2 (5.3%) as simple epithelial cysts, 2 (5.3%) as cystadenocarcinomas (Figure 3), 2 (5.3%) as mucinous cystadenomas, and 1 (2.6%) as a serous cystadenoma (Figure 4).

As can be seen in Table 2, neoplastic cysts were more prevalent in female patients (seen in 60.5%) and non-neoplastic cysts were more prevalent in male patients (seen in 75%). Among the patients with neoplastic cysts, 73.7% were over 60 years of age, whereas 75.0% of the patients with pseudocysts were between 40 and 59 years of age. Half of the patients had a solitary cyst, and 39.5% had three or more cysts. The majority of the cysts were between 10 mm and 30 mm in diameter and were located in the pancreatic head, with no calcifications, parenchymal atrophy, ductal dilation, or solid components.

**Table 2:** Characteristics of patients with neoplastic and non-neoplastic pancreatic cysts.

	Neoplastic cyst	Pseudocyst	
Characteristic	(n = 38)	(n = 4)	*P*-value
Gender			
Male	15 (39.5%)	3 (75%)	0.297
Female	23 (60.5%)	1 (25%)	
Age group			
18-39 years	1 (2.6%)	0	0.096
40-59 years	9 (23.7%)	3 (75%)	
> 60 years	28 (73.7%)	1 (25%)	
Cyst location within the pancreas			
Head	14 (36.8%)	1 (25%)	0.958
Body	8 (21.2%)	1 (25%)	
Tail	4 (10.5%)	0	
Multiple	12 (31.5%)	2 (50%)	
Parenchymal atrophy			
Yes	2 (5.3%)	0	0.221
No	36 (94.7%)	4 (100%)	
Number of cysts			
1	19 (50%)	2 (50%)	0.836
2	4 (10.5%)	1 (25%)	
≥ 3	15 (39.5%)	1 (25%)	
Diameter of the largest cyst			
< 10 mm	16 (42%)	1 (25%)	0.100
10-30 mm	18 (47.5%)	1 (25%)	
> 30 mm	4 (10.5%)	2 (50%)	
Parietal calcification			
Yes	0	0	—
No	38 (100%)	4 (100%)	
Ductal dilation			
Yes	9 (23.7%)	0	0.272
No	29 (76.3%)	4 (100%)	
Solid component			
Yes	3 (7.9%)	1 (25%)	0.268
No	35 (92.1%)	3 (75%)	

**Figure 5:**
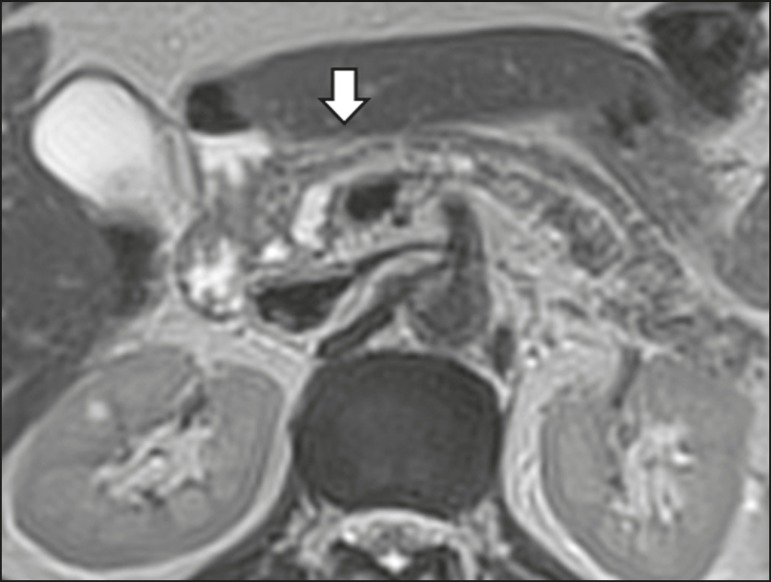
T2-weighted MRI sequence showing dilation of the pancreatic duct (arrows) caused by a cyst.

Among the 38 patients diagnosed with neoplastic cysts, 10 (26.3%) presented radiological signs of malignancy risk (Table 3): 9 (23.7%) with ductal dilation (Figure 5); 3 (7.9% ) with a solid component (Figure 3); and 3 (7.9%) that were larger than 30 mm (Figure 3). The prevalence of risk factors was higher among male patients (40.0%), among patients who were over 60 years of age (32.1%), and among patients with three or more cystic lesions (40.0%).

**Table 3:** Association between neoplastic cysts and radiological signs of malignancy risk (ductal dilation and/or cyst diameter > 30 mm and/or cyst with a solid component).

	With signs	Without signs	
	of malignancy	of malignancy	
Variable	risk* (n = 10)	risk* (n = 28)	*P*-value
Gender			
Male	6 (40%)	9 (60%)	0.150
Female	4 (17.4%)	19 (82.6%)	
Age group			
18-39 years	0	1 (100%)	0.383
40-59 years	1 (11.1%)	8 (98.9%)	
> 60 years	9 (32.1%)	19 (67.9%)	
Cyst location within the pancreas			
Head	2 (14.3%)	12 (85.7%)	0.473
Body	2 (25%)	6 (75%)	
Tail	1 (25%)	3 (75%)	
Multiple	5 (41.7%)	7 (58.3%)	
Number of cysts			
1	3 (15.8%)	16 (84.2%)	0.281
2	1 (25%)	3 (75%)	
≥ 3	6 (40%)	9 (60%)	

* Ductal dilation and/or cyst diameter > 30 mm and/or cyst with a solid component.

## DISCUSSION

Pancreatic cystic lesions have been identified during imaging examinations with increasing frequency, mainly due to the dissemination of sectional imaging methods. The incidence of pancreatic cystic lesions in the general population of the United States is estimated to be between 3% and 15%, the prevalence increasing with age^(11)^. The present study evaluated the prevalence of pancreatic cystic lesions in a population of adults undergoing CT and MRI examinations, as well as its association with risk factors for malignancy.

Cystic lesions of the pancreas were identified in 4.5% of the patients, being detected more often by MRI than by CT (in 6.1% vs. 3.1% of the scans). That trend has been confirmed by other authors, such as Laffan et al.^(12)^, who reported a prevalence of 2.6% on CT scans, and Zhang et al.^(13)^, who reported a prevalence of 19.6% on MRI scans. That discrepancy might be related to differences in accuracy among the diagnostic methods employed or to population differences, given that other studies have show that MRI is slightly more sensitive than is CT for the diagnosis of pancreatic cystic lesions^(10,14,15)^.

CT and MRI have specific characteristics in the imaging evaluation of the abdomen. CT has broad utility in the evaluation of abdominal complaints and currently functions as a screening test for various diseases, playing a prominent role in the evaluation of emergency or nonspecific abdominal pain. MRI of the abdomen provides additional information, combining anatomical and functional images within specific study protocols for various clinical indications; it represents a more selective imaging modality, with a longer acquisition time, and is usually requested on an outpatient basis by specialists in abdominal diseases. Those characteristics can influence the epidemiological profile of the patients submitted to imaging examinations of the abdomen and could have contributed to the higher rate of detection of pancreatic cystic lesions by MRI. In addition, MRI presents better tissue characterization and better identification of small cystic components.

In the present study, the prevalence of pancreatic cystic lesions in patients with pancreatic complaints was 42.9%, approximately 17 times higher than in patients with nonpancreatic complaints. That might be explained by the fact that some patients were being followed because of a previous diagnosis of cystic lesions or were already under suspicion of having pancreatic disease. We found no significant difference between genders in terms of the prevalence of neoplastic cysts. However, the prevalence of pseudocysts was three times higher in males, which could be explained by the prevalence of alcoholism, which is the main cause of chronic pancreatitis in Brazil^(16)^, is higher among males.

The mean age of patients with cysts was 67.9 years, and the prevalence of pancreatic cystic lesions increased linearly with age, reaching 7.6% in patients over 60 years of age (*p* < 0.01). These findings are similar to those described by other authors^(10,13,17)^.

Pancreatic cystic lesions comprise a broad spectrum of diseases that can be categorized into two groups^(11,18)^: non-neoplastic cysts (traumatic, inflammatory, or congenital in origin); and neoplastic cysts, including serous cystadenoma, mucinous cystadenoma, intraductal papillary mucinous neoplasm, solid-cystic pseudopapillary tumor, cystic neuroendocrine tumor, and cystadenocarcinoma. The imaging characteristics can facilitate the etiological diagnosis and therapeutic planning, given the highly varied clinical spectrum and prognosis of these lesions^(19)^.

In the present study, cysts were classified as neoplastic or non-neoplastic according to their morphological characteristics on CT and MRI, as well as clinical findings. Of the 42 cystic lesions identified, 4 (9.5%) were classified as non-neoplastic cysts (pseudocysts) and 38 (90.5%) were classified as neoplastic cysts. Pseudocysts were characterized as pancreatic cystic lesions without solid components, associated with peripancreatic inflammatory changes in patients with a history of pancreatitis^(11)^.

Most of the neoplastic cystic lesions were characterized as intraductal papillary mucinous neoplasms (81.6%), with few cases of simple epithelial cyst (in 5.3%), cystadenocarcinoma (in 5.3%), mucinous cystadenoma (in 5.3%), and serous cystadenoma (in 2.6%). These findings represent only the radiological impression, without histopathological confirmation. However, they suggest that, among incidental imaging findings in the pancreas, benign cystic lesions predominate. In a cross-sectional population-based study conducted in the United States between 2005 and 2009, Gardner et al.^(6)^ detected a prevalence of pancreatic cysts of 2.5% in the adult population, the frequency of adenocarcinomas among patients with pancreatic cysts being 0.03%. Other authors have reported that the increase in the rate of detection of intraductal papillary mucinous neoplasms in imaging studies has not translated to an increase in the mortality associated with this neoplasm or in the overall mortality related to pancreatic cancer, thus likely resulting in an increase in diagnostic monitoring and not in the number of patients diagnosed with clinically relevant disease^(20)^.

Intraductal papillary mucinous neoplasm usually presents as a cystic lesion in contact with the main pancreatic duct or secondary ducts. It can have a multilocular appearance, with a solid component, accompanied by ductal dilation and parenchyma atrophy^(19,21)^. Mucinous cystadenomas present as unilocular cystic lesions, with walls well differentiated from the rest of the pancreatic parenchyma, and can be divided into multiple compartments by thin septa, with or without thick contents (mucin). Such lesions have a radiological appearance similar to that of pancreatic pseudocysts, and the clinical history plays a fundamental role in their differentiation. Serous cystadenomas appear as lesions that are well delimited by a fibrous capsule, containing numerous microcysts with fluid content, in a honeycomb aspect, in some cases being macrocystic and unilocular^(11,19)^.

Cystadenocarcinoma usually presents as an infiltrative, hypovascular mass, with obstruction of the pancreatic duct and invasion of adjacent vascular structures. In rare cases, the cystic component can be identified by imaging examination and is associated with necrosis or retention of pancreatic secretion due to ductal obstruction^(21)^. Albeit uncommon, cystadenocarcinoma has an exceptionally high mortality rate, making it one of the most common causes of cancer mortality in developed countries. Chernyak et al.^(22)^ found that patients with pancreatic cystic lesions are at an increased risk of developing adenocarcinoma. According to the guidelines of the American Gastroenterology Association^(11)^, the imaging findings that are predictive of malignancy in pancreatic cystic lesions are cyst size, ductal dilation, and the presence of a solid component.

Various authors have analyzed pancreatic cysts after surgical resection and have shown that lesions larger than 30 mm in diameter were malignant twice as often as were those with a diameter smaller than 30 mm, that cut-off point being found to have a sensitivity of 68-80% and a specificity of 44-54%. Cystic lesions with a solid component have been found to be malignant three times more often than are those without, that factor being found to have a sensitivity of 42-54% and a specificity of 88-93%. Likewise, the incidence of malignancy is approximately 50% higher for cystic lesions with ductal dilation than for those without, with a sensitivity of 25-38% and a specificity of 75-84%^(11)^.In the present study, 10 (26.3%) of the 38 patients diagnosed with neoplastic cysts had risk factors for malignancy (diameter > 30 mm, presence of a solid component, or ductal dilation): 9 (23.7%) with ductal dilation; 3 (7.9%) with a solid component; and 3 (7.9%) with a diameter > 30 mm. Of the cases with risk factors, 32.1% were in patients over 60 years of age, 40.0% were in males, and 60.0% were in patients with more than one cystic lesion. In the present study, neither clinical features nor morphological aspects showed a statistically significant association with neoplastic cysts or warning signs for malignancy. That might be attributable to the fact that the final number of pancreatic cystic lesions evaluated was relatively small and therefore did not allow us to draw definitive conclusions.

Our study included a large population undergoing imaging examinations, in which the detection of cystic lesions of the pancreas was similar to patterns described in the literature. However, this study has some limitations. The patients had been seen at a single imaging center, where the outpatient protocols were unaffiliated with any hospital and thus might have reflected the epidemiological characteristics of the population. The predominance of patients with private health insurance plans might also reflect the socioeconomic level of the sample. In some cases, there was little clinical information limiting the rationale for radiological diagnosis. The fact that all of the cases were reviewed by the same radiologist guaranteed homogeneous criteria for the classification of the pancreatic cystic lesions detected in this study, although it precluded the analysis of variations between observers with different levels of experience. Long-term follow-up of patients undergoing imaging examinations could facilitate the diagnostic elucidation of the cystic lesions detected.

In conclusion, the prevalence of pancreatic cystic lesions detected by CT and MRI in the present study was 4.5%, such lesions being more common in patients over 60 years of age or with any pancreatic complaint. The rate of cystic lesion detection was higher for MRI. Most of the lesions presented features characteristic of intraductal papillary mucinous neoplasm. Signs of malignancy risk were observed in 26.3% of the neoplastic cysts detected.
